# Genomic diversity of SARS-CoV-2 in Malaysia

**DOI:** 10.7717/peerj.12449

**Published:** 2021-11-03

**Authors:** Noorliza Mohamad Noordin, Joon Liang Tan, Chee Kheong Chong, Yu Kie Chem, Norazimah Tajudin, Rehan Shuhada Abu Bakar, Selvanesan Sengol, Hannah Yik Phing Phoon, Nurul Aina Murni Che Azid, W Nur Afiza W Mohd Arifin, Zirwatul Adilah Aziz, Hani Hussin, Nurul Syahida Ibrahim, Aziyati Omar, Ushananthiny Ravi, Kamal Hisham Kamarul Zaman, Mohd Asri Yamin, Yun Fong Ngeow

**Affiliations:** 1 National Public Health Laboratory, Ministry of Health Malaysia, Sungai Buloh, Selangor, Malaysia; 2 Faculty of Information Science and Technology, Multimedia University, Melaka, Melaka, Malaysia; 3 Public Health Department, Ministry of Health, Putrajaya, Malaysia; 4 Faculty of Medicine and Health Sciences, Universiti Tunku Abdul Rahman, Bandar Sungai Long, Selangor, Malaysia; 5 Center for Research on Communincable Diseases, Universiti Tunku Abdul Rahman, Bandar Sungai Long, Selangor, Malaysia

**Keywords:** SARS-CoV-2, Genetic diversity, Lineages, Mutations, Linkage disequilibrium

## Abstract

**Background:**

More than a year after its first appearance in December 2019, the COVID-19 pandemic is still on a rampage in many parts of the world. Although several vaccines have been approved for emergency use, the emergence and rapid spread of new SARS-CoV-2 variants have sparked fears of vaccine failure due to immune evasion. Massive viral genome sequencing has been recommended to track the genetic changes that could lead to adverse consequences.

**Methods:**

We sequenced SARS-CoV-2 respiratory isolates from the National Public Health Laboratory, Malaysia and examined them together with viral genomes deposited in GISAID by other Malaysian researchers, to understand the evolutionary trend of the virus circulating in the country. We studied the distribution of virus lineages and site-wise mutations, analysed genetic clustering with the goeBURST full Minimum Spanning Tree algorithm, examined the trend of viral nucleotide diversity over time and performed nucleotide substitution association analyses.

**Results:**

We identified 22 sub-lineages, 13 clonal complexes, 178 sequence types and seven sites of linkage disequilibrium in 277 SARS-CoV-2 genomes sequenced between January and December 2020. B.1.524 was the largest lineage group. The number of mutations per genome ranged from 0 to 19. The mean genomic diversity value over 12 months was 3.26 × 10^−4^. Of 359 mutations detected, 60.5% of which were non-synonymous, the most frequent were in the *ORF1ab* (P4715L), *S* (D614G and A701V) and *N* (S194L) genes.

**Conclusion:**

The SARS-CoV-2 virus accumulated an abundance of mutations in the first year of the COVID-19 pandemic in Malaysia. Its overall genetic diversity, however, is relatively low compared to other Asian countries with larger populations. Continuous genomic and epidemiological surveillance will help to clarify the evolutionary processes determining viral diversity and impacting on human health.

## Introduction

Despite world-wide control efforts, the COVID-19 pandemic continues to ravage many populations since it was first reported in December 2019. This protraction has been attributed partially to the emergence of SARS-CoV-2 mutants that have been linked with increased infectivity and the ability to resist host immune responses. The best known variants with global spread since their introduction include the UK variant B.1.1.7 N501Y that has been shown to have higher transmissibility and risk of death than the wild-type virus (Challen et al., 2021), the S. African variant B.1.351 20H/501Y.V2 with spike protein mutations that apparently led to more frequent and more serious infections among young adults with no underlying illnesses and decreased neutralisation by antibodies (Planas et al., 2021) and the Brazilian variant P.1 GR/501Y.V3 with unique mutations in the receptor binding domain of the spike protein that has been linked with greatly increased transmissibility, higher mortality and lower susceptibility to inactivation by anti-S antibodies (Planas et al., 2021).

In South-East Asia, Malaysia is one of the countries to report COVID-19 infections as early as January 2020 when a few cases were imported from China. Since then, local transmission and further importation from different parts of the world increased the number of confirmed cases to 115,078 (an incidence of 352.14/100,000) with 474 (0.41%) deaths by 1 January 2021 and 514 clusters of infection reported in the 12-month period (Fun et al., 2021). One of the largest was associated with a Tablighi Jamaat religious mass gathering held in Kuala Lumpur between February 27 and March 3, 2020, that was attended by about 16,000 people (10% foreigners from many different countries) and 34 deaths among 3,375 infected individuals (Fun et al., 2021). From this event, the infection was spread all over Malaysia and to neighbouring countries as well. Other prominent outbreaks included the Pesantren cluster in April that was linked to Malaysian students returning from a religious school in East Java, Indonesia, and the Sivaganga cluster in July-August that was traced to a Malaysian who returned from India carrying the D614G variant (Danial et al., 2020). Non-Malaysians were predominant in a few clusters among construction workers and inmates of prisons and immigration detention centres. From October 2020 onwards, increasing cases occurred in the East Malaysian states of Sabah and Sarawak causing the incidence and the effective reproduction number (Rt) of COVID-19 there to exceed those in Peninsular Malaysia (WHO, 2021). Up to April 2021, five infections by B.1.1.7 N501Y and 21 by B.1.351 20H/501Y.V2 were reported by the Ministry of Health, Malaysia (Malaymail, 2021a; Malaymail, 2021b).

In this paper, we analysed 277 genome sequences from Malaysian SARS-CoV-2 isolates to study the diversity of the viral genomes over time, and to monitor the emergence of mutations that could affect the ability of the virus to spread or to cause more severe illness. We specifically looked out for rapidly spreading mutants that have emerged in other countries.

## Methodology

### Ethics statement

This study received waiver on informed consent by the Ministry of Health Medical Research Ethics Committee (no. KKM/NIHSEC/ P20-1094).

### Source of SARS-CoV-2 genomes

The 277 SARS-CoV-2 genomes analysed comprised 30 sequenced (Table S1) by the National Public Health Laboratory Malaysia (MKAK) and 247 lodged in GISAID (Elbe & Buckland-Merrett, 2017) by other Malaysian research institutions, namely, the Institute for Medical Research (IMR), the Malaysian Genome Institute (MGI) and public universities (University Malaya Medical Centre and University Malaya Pahang). The genomes downloaded from the GISAID database were accessed on 5 January 2021. They represented 421 genomes deposited at different time points in Malaysia throughout the year 2020.

At the MKAK, nasopharyngeal and oropharyngeal swabs from patients seen in public hospitals were used for virus isolation in Vero E6 cells. From each patient, the isolate from the earliest available sample during illness and yielding the highest viral load was selected for whole genome sequencing using the MiSeq platform. The sequencing reads were evaluated in FastQC (Andrews, 2010) and the data obtained were preprocessed in PRINSEQ (Schmieder & Edwards, 2011), according to the quality assessments in the FastQC. In brief, the sequences were quality processed with phred probability mean score of Q20, 5′ and 3′ ends clipping, reads deduplication, removal of reads containing ambiguous nucleotide and removal of trimmed reads <67 bp. The preprocessed data were subjected to assembly in MEGAHIT (Li et al., 2015).

### Genome analysis

All 421 sequenced and downloaded genomes were screened for bases other than IUPAC non-ambiguous nucleotides. This process resulted in 277 genomes for comparisons. All these genomes were aligned against Wuhan-Hu-1(NCBI Accession: NC_045512.2; GISAID Accession: EPI_ISL_402125) in MAFFT (Katoh et al., 2002). The mutations and corresponding amino acid changes were evaluated manually in MEGA X (Kumar et al., 2018). The clonal compositions of SARS-CoV-2 were evaluated with Minimum-Spanning Tree of Phyloviz 2.0 (Francisco et al., 2012). All genomes that emerged from a single founder were considered as belonging to a single cluster. Genomic and gene diversity studies were performed in DNASP 6 (Rozas et al., 2017). Additionally, DNASP 6 was further utilized to infer linkage disequilibrium which is the degree of non-random association of alleles (D′). Besides allele frequency, the linkage disequilibrium was calculated by considering the correlation coefficient of variant frequency (r^2^) (VanLiere & Rosenberg, 2008). A strong association of mutations was predicted by an r^2^ value > 0.8 and a Fisher one-tailed test of significance value < 0.01. All analyses were conducted with default parameters.

## Results

The genome analysis results based on Pangolin COVID-19 Lineage Assigner (https://pangolin.cog-uk.io/) showed 22 lineages with the lineage group B.1.524 forming the majority (107, 38.6%), followed by B.6 (41, 14.8%), B (22, 7.9%), B.1.129 (21, 7.6%), B.6.1 (20, 7.2%) and other smaller groups (Fig. S1). All genomes were aligned at least 99% against the Wuhan-HU-1 genome except for two with only approximately 94% alignment.

### Mutations in SARS-CoV-2 from Malaysia

Of the 277 genomes that underwent genome-wide alignment, nine were found to be identical to Wuhan-Hu-1. Of these, four were from Malaysian patients with no history of travel outside the country.

Viewing site-wise mutations, a total of 359 positions with variants were predicted, 63.8% of which were found to be solitary across the genomes analysed. These mutations were observed within nine protein encoding genes and four non-coding sites. Approximately 60% of mutations were non-synonymous and 67% involved a base substitution to Thymine. The highest number of mutations predicted in one genome was 19.

The most frequently seen mutations are listed in Table 1. Those shared by at least 100 genomes were at 13 genome positions (241, 3037, 6312, 8637, 10124, 14408, 17518, 21516, 21622, 23403, 23664, 28133, and 28854) with eight non-synonymous mutations in the *ORF1ab* (T2016I, T2791I, T3287A, P4715L, L5752F), *S* gene (D614G, A701V) and the *N* gene (S194L) respectively. The mutations shared by 50–99 genomes were at five positions (6312, 11083, 13730, 23929, and 28311). The non-synonymous mutations were in the *ORF1ab* (T2016K, L3606F, A4489V) and *N* (P13L). At the 6312 position there were two mutations, one with a T2016I (C- > T) change that was shared by >100 genomes and a second with a T2016K (C- > A) change that was shared by only 85 genomes. Excluding the substitution at position 241 in the 5′ UTR, the top three most frequent mutations were the C3037T (59.9%), C14408T (59.9%) and A23403G (D614G) (57.4%). These three mutations were reported to be found in SARS-CoV-2 genomes worldwide SARS-CoV-2 genomes worldwide.

**Table 1:** Mutations in at least 50 genomes of SARS-CoV-2.

**Nucleotide substitution position**	**Impact of mutation**	**Gene**	**Number of strains**
241	–	5′UTR	162
3037	Synonymous	*ORF1ab*	168
6312	T2016I	*ORF1ab*	106
8637	T2791I	*ORF1ab*	106
10124	T3287A	*ORF1ab*	107
14408	P4715L	*ORF1ab*	166
17518	L5752F	*ORF1ab*	106
21516	Synonymous	*ORF1ab*	107
21622	Synonymous	*S*	107
23403	D614G	*S*	159
23664	A701V	*S*	107
28133	Synonymous	*ORF8*	107
28854	S194L	*N*	112
6312	T2016K	*ORF1ab*	85
11083	L3606F	*ORF1ab*	91
13730	A4489V	*ORF1ab*	87
23929	Synonymous	*S*	81
28311	P13L	*N*	82

Less frequent mutations (Table S2) included a unique 2 bp-deletion in *ORF8* (genomic position 28066–28067) that was found in only one genome, and the mutations (C6310A, T7621C and C19524T) reported by neighbouring countries such as Singapore, Australia, and India (Chong et al., 2020). There was only one genome each with the T8782C and C28144T mutations that have been used to distinguish the type B variant predominant in East Asia from the type A variant predominant in North America and Europe (Forster et al., 2020)

### Identification of super spreader mutations

We specifically looked for mutations that have been linked to increased transmission in various countries. As shown in Table 1 above, the D614G amino acid substitution in the S spike protein was observed in 159 of 277 (57.4%) genomes. Based on the isolation dates in GISAID, this mutation was present in the viruses isolated in March 2020, suggesting that D614G was present in Malaysia before it was first reported in the Sivaganga cluster in July-August 2020. None of our D614G mutations were accompanied by the S477N substitution noted to be frequently alongside D614G in the S protein (Singh et al., 2021). We did not find the genomes of the UK, S. African and Brazilian super spreaders, although the E: P71L and S: A701V mutations defining the S. African variant B.1.351 501Y.V2 were found separately, in one and 107 genomes, respectively.

### Clonality and diversity of SARS-CoV-2 genomes

**Figure 1:**
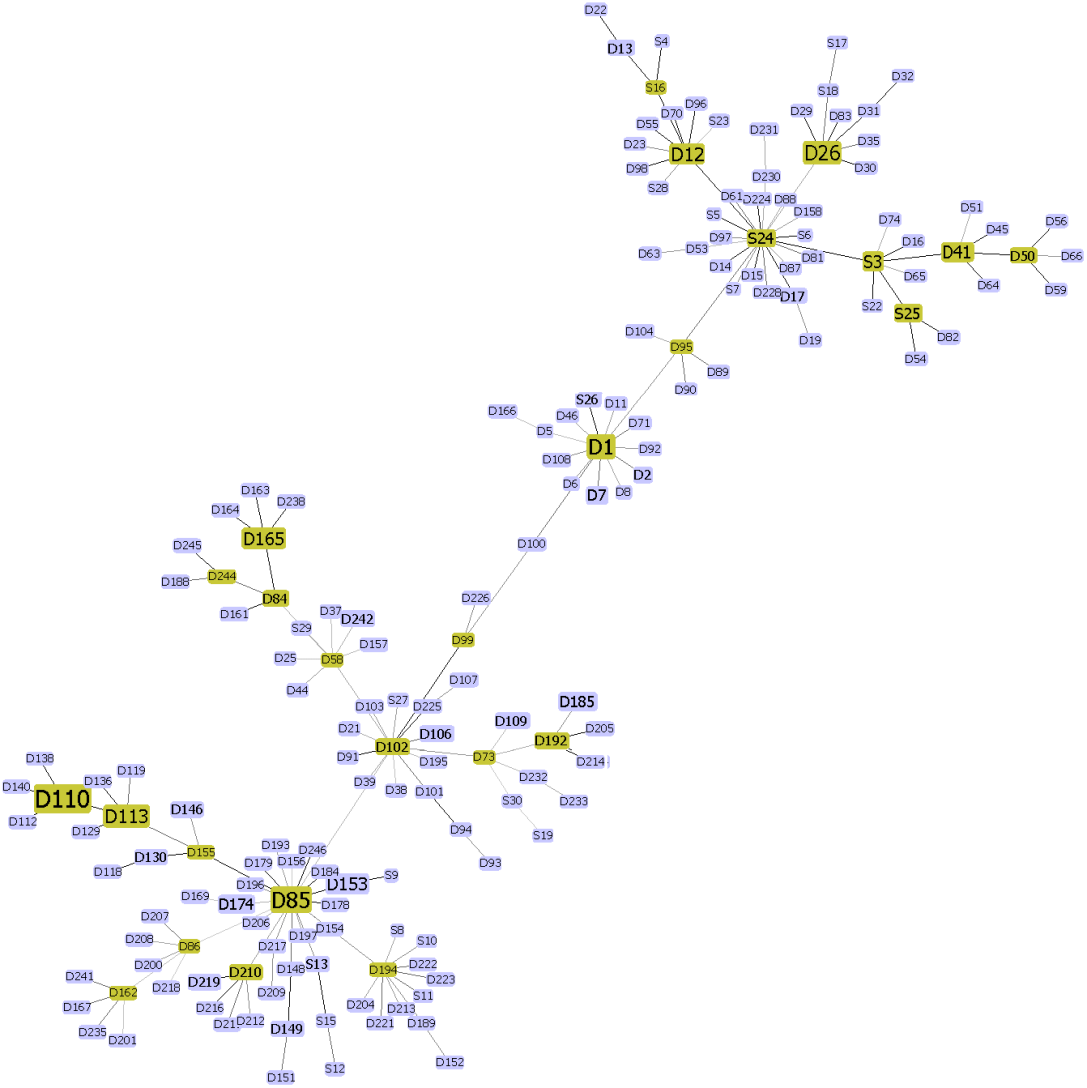
Diversity illustration of SARS-CoV-2 in Malaysia generated in Minimum Spanning Tree (MST). Each node represents either a strain or group of strains and linked based on MST. “D#” denotes downloaded genome and “S#” denotes genome sequenced in this study.

The genomic variations of the 277 SARS-CoV-2 genomes were summarized with the goeBURST full Minimum Spanning Tree algorithm designed to detect clusters defined by genetic sequence similarities (Fig. 1). The analysis indicated 115 groups, of which 13 formed clonal complexes. There were 178 sequence types (STs) with 26 being subgroup founder’s STs. The clonal complexes were not related to the month of virus isolation, or to other epidemiological features described for the outbreaks in different communities.

**Figure 2:**
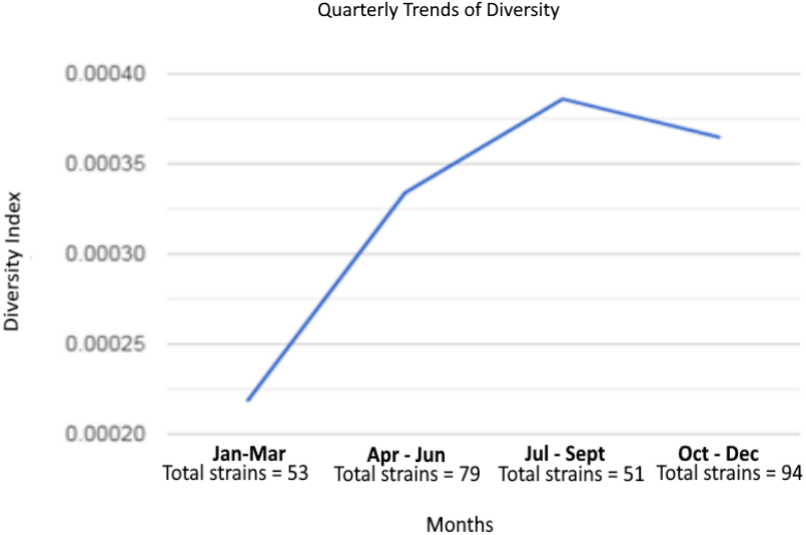
Graph of SARS-CoV-2 diversity in Malaysia in 2020.

We further evaluated the trend of diversity in the SARS-CoV-2 genomes (Fig. 2). In 2020, the viral genomic diversity (the average number of nucleotide differences per site between sequences and its sampling variance) increased from 2.18 × 10^−4^ in the first quarter of the year to 3.34 × 10^−4^ in the second quarter and 3.86 × 10^−4^ in the third quarter before it fell to 3.65 × 10^−4^ in the final quarter. The mean genomic diversity was 3.26 ×10^−4^.

Although the number of viral genomes analyzed in this study was small, the changes in diversity reflected major events in the country. Early in 2020, the diversity value was low because there were only a few viruses in circulation. A steep incline in diversity soon occurred with the spread of infections and hence opportunities for mutations to occur in the local population, and further importation from other countries which would have brought in different lineages and genetic variants of the SARS-CoV-2 virus. From August to December, although the number of reported cases increased, virus diversity showed a decline. The reason could be that the genomes we analyzed were mostly from a few large clusters and did not include new cases responsible for the spike in infections in Sabah towards the end of the year, as there were no SARS-CoV-2 genomes from Sabah in GISAID at the time we accessed the database.

**Figure 3:**
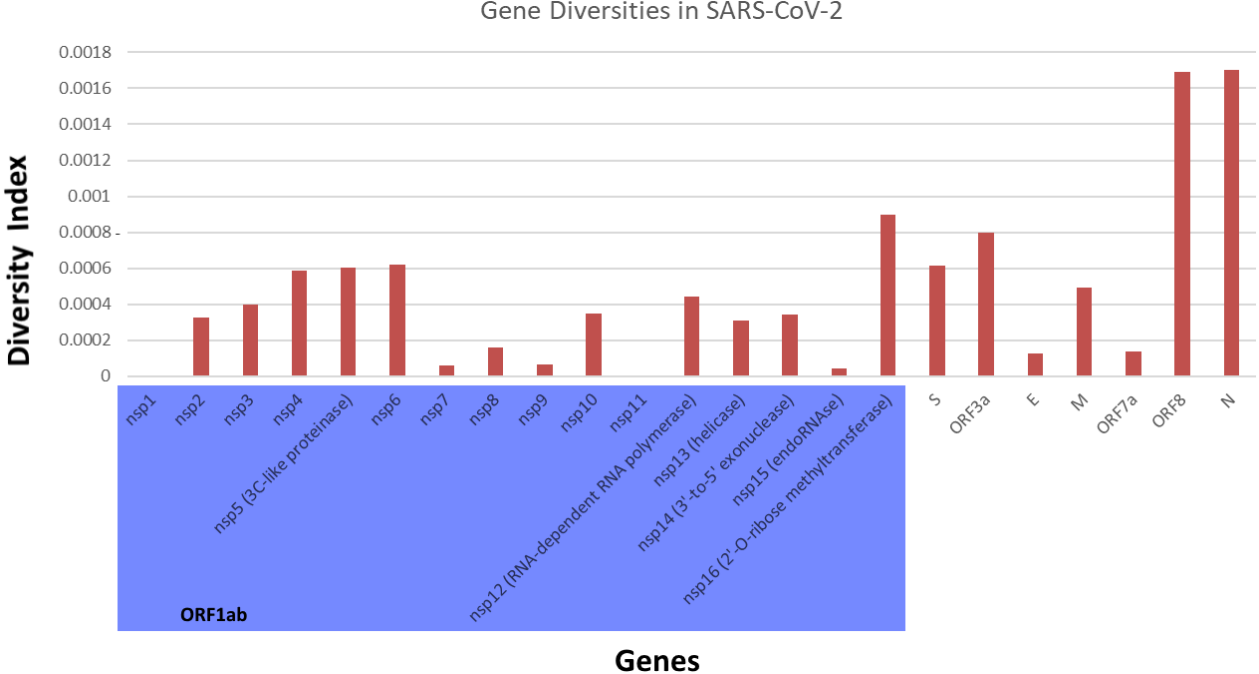
Gene Diversity of SARS-CoV-2 in Malaysia.

To study nucleotide diversity of the SARS-CoV-2 genes affected by mutations, we looked at the genes for the four structural proteins E (envelope), M (membrane), N (nucleocapsid), S (spike), and the ORFs (open-reading frames). Figure 3 shows the diversity indices of eight genes which were, in descending order, 1.7 × 10^−3^ (N), 1.69 × 10^−3^ (*ORF8*), 0.79 × 10^−3^ (*ORF3a*), 0.61 ×10^−3^ (*S*)*,* 0.49 ×10^−3^ (*M*), 0.41 ×10^−3^ (*ORF1ab*), 0.14 × 10^−3^ (*ORF7a*) and 0.13 ×10^−3^ (*E*). The *N, E, S* and *ORF1ab* are genes often used in RT-PCR assays for the diagnosis of COVID-19. A high diversity in the N gene is expected to decrease its sensitivity in these assays (Hasan et al., 2021). The high diversity in *ORF8* is consistent with reports describing it as a rapidly evolving gene (Ceraolo & Giorgi, 2020). Similarly, the lower diversity values in the *M* and *ORF7a* genes are not surprising as these genes have been reported to be stable genes that are suitable targets for vaccine development (Nguyen et al., 2020). There were 16 non-structural proteins (nsp) coded in *ORF1ab*. Both *nsp1* and *nsp11* showed a diversity index of 0, indicating high nsp conservation in the genomes. The most diverse nsp was *nsp16* (diversity of 8.99 ×10^−4^). The diversity indices of the remaining nsp ranged from 4.47 ×10^−5^ to 6.17 ×10^−4^.

### Nucleotide substitution association analyses

**Figure 4:**
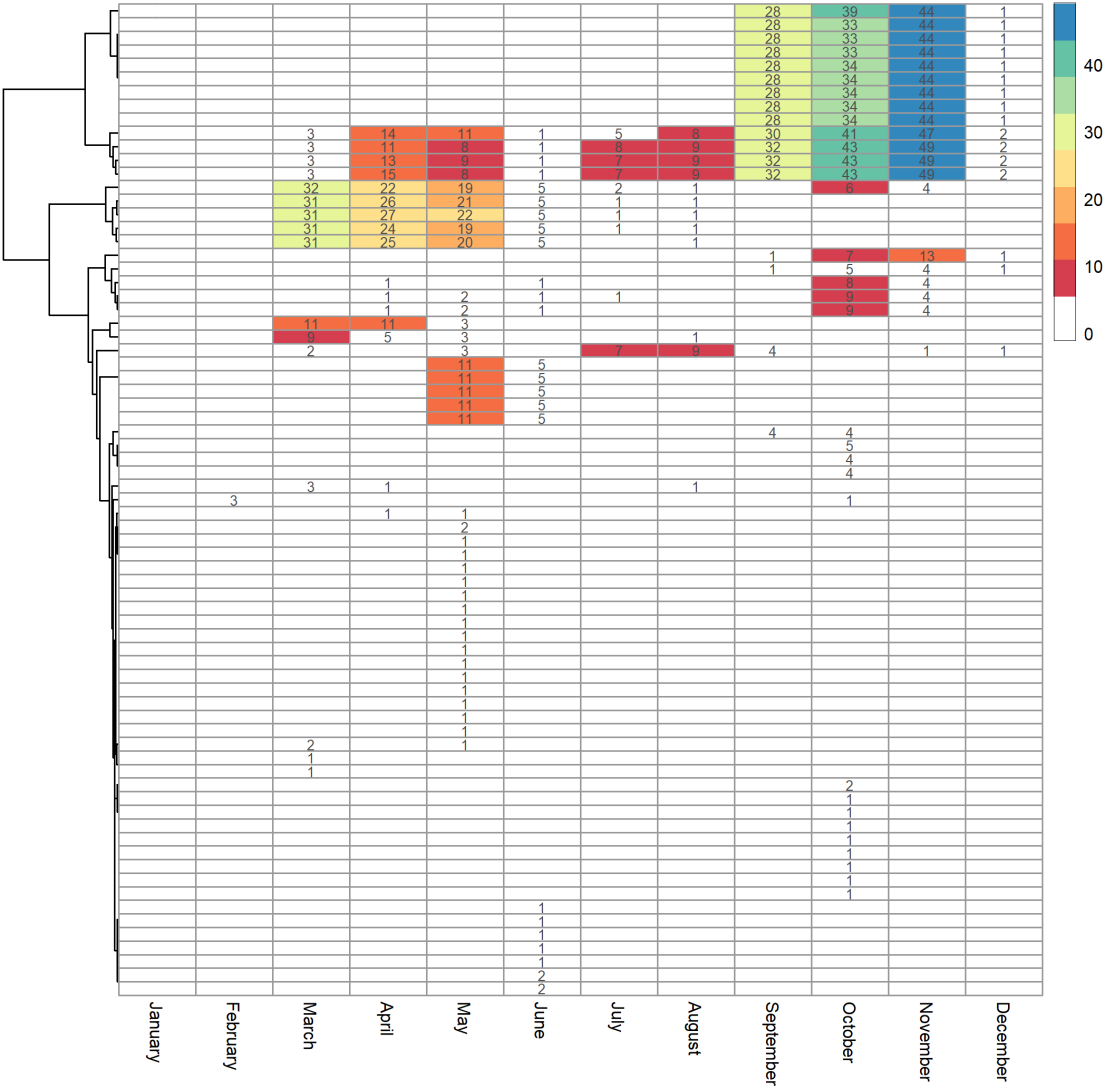
Heatmap of mutations in SARS-CoV-2 against months. The number in each cell indicates the number of strains having the mutation.

The temporal study on viral mutations showed that in January and February 2020, there were only a few mutations. From March to December, substitutions appeared consistently at the genomic positions C241T, C3037T, C14408T and A23403G. In addition, 10 more mutations appeared from September to December. These mutations were C5869T, C8637T, A10124G, C17518T, C21365T, C21516T, C21622A, C23664T, A28133T and C28854T. Two mutations, C13730T and C23929T were noted to be present in viruses isolated in March to August. At the genomic position of 6312, one mutation was observed in March to August (C6312A) and another appeared from September to December (C6312T). Month-specific mutations were the highest in May and not in November and December, the two months with the largest number of reported cases (Fig. 4).

When we checked for non-random mutational associations, we hypothesized that non-random association of mutations should be present in accordance with the frequency of occurrence. For this, we inferred based on r^2^ and supported with Fisher one-tailed test of significance on the parsimony informative sites. The analyses returned seven sets of non-redundant site comparisons above the threshold with the r^2^ ranging from 0.8 to 1.0 and with coefficient of linkage disequilibrium (LD) ranging from 0.93 to 1.0 (Table S3). All the positions identified were supported with a *p*-value of 0. The first LD set was observed in the viruses isolated in October 2020, at the genomic positions of A1904G, C12488T and G23236T. They were all non-synonymous mutations in *ORF1ab*. The second LD set comprised C3037T, C14408T and A23403G mutations in viruses isolated from March to December and involving a single synonymous and non-synonymous mutation in *ORF1ab*, together with a single non-synonymous mutation in the S protein. These are the mutations also reported by others (Rouchka, Chariker & Chung, 2020) to be linked. The third LD set was found in eight genomic positions, namely C8637T, A10124G, C17518T, C21516T, C21622A, C23664T, A28133T and C28854T. Except for C21516T, C21622A and A28133T, all were non-synonymous mutations, spanning *ORF1ab*, S protein, *ORF8* and N protein in viruses isolated from September to December 2020. The fourth LD set involved four mutations across three different proteins, at the genomic positions of G11083T, C13730T, C23929T and C28311T. The mutations were synonymous in the S protein, non-synonymous in N protein and non-synonymous in two positions in the ORF1ab. The fifth LD set involved genomic positions C13329T, C20823T, C26607T and A29086T. C20823T and A29086T caused non-synonymous mutations in the *ORF1ab* and *N* genes respectively, in viruses isolated in May and June. In the sixth LD set, C18877T, C26735T and G25563T were observed, the former two as synonymous mutations in *ORF1ab* and M protein, respectively, and the third as a non-synonymous mutation in *ORF3a*. Lastly, LD was observed in C24382T and G28307T causing a synonymous mutation in the S protein, and a non-synonymous mutation in the N protein, respectively. All in, the LD sets involved 21 non-synonymous and eight synonymous mutations in the *ORF1ab, ORF8, ORF3a, M, N* and *S* genes. While the LD sets were observed to be associated with the month of virus isolation, they were not clustered in the Minimum Spanning Tree.

## Discussion

The World Health Organisation has strongly advocated routine genomic surveillance for the SARS-CoV-2 virus (WHO, 2021). A principal aim is to look out for new variants that might cause adverse consequences such as increased viral infectivity and virulence, resistance to therapeutic agents, and immune evasion. As countries world-wide adopt vaccination to end the COVID-19 pandemic, many fear that under vaccine pressure, SARS-CoV-2 variants will emerge with new strategies to escape vaccine-induced immunity.

The diversity of SARS-CoV-2 is analysed to understand its pathogenicity, origin, and evolutionary implications (Forster et al., 2020; Parlikar et al., 2020). In this study, we found a mean genomic diversity index of 3.26 ×10^−4^ for Malaysian viruses sequenced between January and December 2020. This value is lower than those reported by Flores-Alanis et al. (2021) for Asia (4.2 ×10^−4^) and six other geographical regions in the world (0.44 × 10^−4^). The generally low diversity in the SARS-CoV-2 virus has been noted by other researchers (Dearlove et al., 2020; Rauscha et al., 2020) and has been surmised to be due to the virus having an RNA polymerase with proofreading ability to correct errors during RNA synthesis (Ogando et al., 2019) as well as genetic drift and bottleneck events in the evolution of the virus (Rausch, 2020). Although low diversity is currently an advantage for vaccine design, it is expected that this landscape will soon be changed after the world-wide implementation of mass vaccination which would exert a tremendous immune pressure on the evolving virus.

As in most parts of Asia, the B lineage predominated among the 22 lineages identified in our Malaysian viral genomes. The largest lineage group was B.1.524 described in Pango Lineages as a Malaysian lineage that formed 65.0% and 26.0% of SARS-CoV-2 in Malaysia and Singapore, respectively, after it was first reported on 1 Sep 2020 (https://cov-lineages.org/lineages/lineage_B.1.html). Other major lineages (B.6, B.6.1, B.6.2 and B.6.6) were also those originally reported from India, Malaysia, and Singapore, possibly reflecting the frequent population movement among the three countries. There were more non-synonymous than synonymous mutations overall and among the LD sets. Among the mutation hotspots only D614G and A701V in the spike protein as well as P4715L and T2015I in *ORF1ab* are known to have a world-wide prevalence. Only one mutation, a 2 bp-deletion in *ORF8,* is possibly unique (not found among the genome sequences in GISAID).

The pattern of linkage disequilibrium (LD) in a genome is said to reflect the evolutionary history of an organism (Illingworth & Mustonen, 2012). We found seven sets of mutations showing LD, each with at least one non-synonymous mutation, distributed in the *ORF1ab, ORF8, ORF3a, N, M* and *S* genes. One of the LD sets included A23403G (D164G), C14408T and C3037T which were reported to be the most frequent mutations in many parts of the world (Nguyen et al., 2020; Rouchka, Chariker & Chung, 2020) and also found to be the most frequent mutations in this study. The linkage between D641G in the *S* gene and P4715L in *ORF1ab* is consistent with their presence together in >75% of global sequences (Flores-Alanis et al., 2021). While the LD sets were observed to be associated with the month of virus isolation, they were not clustered in the Minimum Spanning Tree, indicating independent evolution of each of the LD sets. As the linkages we observed were also reported by other researchers using different approaches and different cohorts (Rouchka, Chariker & Chung, 2020; Haddad et al., 2021), it is possible that the linked mutations might have evolutionary or survival significance in SARS-CoV-2. However, for most of the LD sets, we could not predict any association between the mutations showing LD and characteristics such as better adaptation to the host or potential for immune evasion.

This study is limited by the small number of SARS-CoV-2 genomes analyzed. Sampling bias could not be ruled out as it was not known how individual researchers selected the viral genomes they deposited in the GISAID database. As the downloaded genomes were only available in the form of assemblies in GISAID, we were unable to evaluate their quality and standardize the data preprocessing with the use of sequencing reads. There was also limited patient and epidemiological data on the genomes we analyzed. Thus, we were unable to discuss issues such as intra-host viral heterogeneity (Tang et al., 2020) or the association of genetic variations with viral infectivity and pathogenicity.

## Conclusions

This sample of SARS-CoV-2 genome sequences showed that within the first year of its appearance in Malaysia, the virus appeared to have accumulated an abundance of mutations including one possibly novel mutation in *ORF8*. Continuous genomic surveillance will provide information on the evolutionary trend of the virus, but adequate coverage of the circulating viruses is necessary to generate truly representative viral genetic characteristics that can be used along with epidemiological data to guide public health control strategies. This would require more extensive genome studies to enable the capture of all important developments in the evolution of the SARS-CoV-2 virus.

## Supplemental Information

10.7717/peerj.12449/supp-1 Supplemental Information 1 Information on 30 Sequenced Genomes

10.7717/peerj.12449/supp-2 Supplemental Information 2 Mutations in lesser than 50 genomes of SARS-CoV-2

10.7717/peerj.12449/supp-3 Supplemental Information 3 List of linkage disequilibrium

10.7717/peerj.12449/supp-4 Supplemental Information 4 Distribution of SARS-CoV-2 lineages included in this study

## Additional Information and Declarations

### DNA Deposition

The following information was supplied regarding the deposition of DNA sequences: The sequenced genomes are available at GISAID: EPI_ISL_1972889 to EPI_ISL_1972895, EPI_ISL_2001114, EPI_ISL_2001115, EPI_ISL_2001213, EPI_ISL_2001429, EPI_ISL_2001430, EPI_ISL_2001481, EPI_ISL_2001482, EPI_ISL_2001643, EPI_ISL_2001644, EPI_ISL_2001645, EPI_ISL_2001654, EPI_ISL_2001655, EPI_ISL_2001656, EPI_ISL_2001659, EPI_ISL_2009116, EPI_ISL_2009193, EPI_ISL_2009232, EPI_ISL_2009233, EPI_ISL_2009234, EPI_ISL_2009305, EPI_ISL_2009529, EPI_ISL_2009530, EPI_ISL_2009595.

### Data Availability

The following information was supplied regarding data availability: The data is available at SRA: SRX11017364 to SRX11017393, SRP321799.
