# Supplementary figures and images for: Genomic diversity of SARS-CoV-2 in Malaysia
Source: PeerJ. 2021 Nov 3;9:e12449. doi: 10.7717/peerj.12449 (PMC8571957; doi:10.7717/peerj.12449)

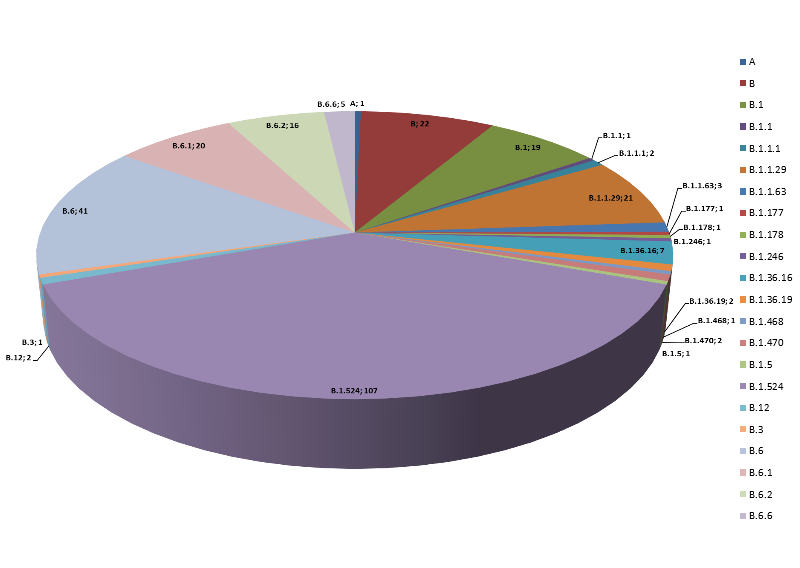

Supplement: Supplemental Information 4 [file peerj-09-12449-s004.png]
